# Cytoscape tools for the web age: D3.js and Cytoscape.js exporters

**DOI:** 10.12688/f1000research.4510.2

**Published:** 2014-10-28

**Authors:** Keiichiro Ono, Barry Demchak, Trey Ideker

**Affiliations:** 1 Department of Medicine, University of California, San Diego, La Jolla, CA 92093-0688, USA

## Abstract

In this paper we present new data export modules for Cytoscape 3 that can generate network files for Cytoscape.js and D3.js. Cytoscape.js exporter is implemented as a core feature of Cytoscape 3, and D3.js exporter is available as a Cytoscape 3 app. These modules enable users to seamlessly export network and table data sets generated in Cytoscape to popular JavaScript library readable formats. In addition, we implemented template web applications for browser-based interactive network visualization that can be used as basis for complex data visualization applications for bioinformatics research. Example web applications created with these tools demonstrate how Cytoscape works in modern data visualization workflows built with traditional desktop tools and emerging web-based technologies. This interactivity enables researchers more flexibility than with static images, thereby greatly improving the quality of insights researchers can gain from them.

## Introduction

Cytoscape was born as a GUI-based, Java desktop application in 2003 ^1, 2^. Today, it is a de-facto standard application for biological network analysis and visualization. Around 2005, Java was one of the dominant programming languages for data visualization applications, and many Java-based feature-rich toolkits were developed ^3^. However, since they were designed before the re-discovery of Ajax ^4^, and JavaScript technology was not mature enough to visualize large scientific data sets at the time, developers could not predict the success of JavaScript and related web technologies today. Cytoscape is still an important platform for biological network data integration and analysis, but for data visualization and sharing, we need a new method to take advantage of modern web technologies. Utilizing HTML5 and other emerging web technologies, the Cytoscape Consortium developed a JavaScript library for network visualization called cytoscape.js ( http://cytoscape.github.io/cytoscape.js/), the successor of Cytoscape Web ^5^, to meet the demand from the Cytoscape user community. Although Cytoscape and Cytoscape.js share some of the core concepts, such as Visual Styles or automatic layouts, they are completely independent software packages and there has been no simple way to use Cytoscape data sets in Cytoscape.js.

Public biological data repositories are still growing rapidly and the demand for visualizing those complex biological data sets is high. Traditionally, analysis and visualization of biological data is done by desktop applications, and in most cases, visualizations created by popular libraries (e.g., matplotlib ^6^, ggplot2 ^7^) are static images. It is hard to perform exploratory data analysis only with static images, especially for large data sets, because some of the details are lost due to the limited size of printed papers or computer screens. For networks, dynamic features, such as zoom or pan, are particularly important because size of all but trivial networks requires such dynamic features to perform meaningful analysis. Instead of developing custom visualization toolkits for specific data sets, the scientific data visualization community is heading towards web-based technologies to utilize actively developed visualization toolkits such as mpld3 ( http://mpld3.github.io/) or Bokeh ( http://bokeh.pydata.org/). D3.js ^8^ is one of the most popular toolkits for creating custom interactive visualizations. If biologists can use the existing powerful desktop application as data authoring tool *and* can publish their results with these emerging web technologies for sharing, it simplifies their workflow for large and complex biological data analysis and visualization.

To bridge the gap between the desktop version of Cytoscape and other web-based data visualization toolkits, we developed Cytoscape modules to generate web-friendly data formats. The goal is to enable shared visualization of Cytoscape networks via a web platform (e.g., browser or HTML5 rendering widgets for tablets). And our strategy is to enable conversion from Cytoscape data objects to a format friendly to web apps, and to provide a template code for creating an interactive web application. In this paper, we present the implementation of Cytoscape data exporters and template web applications and demonstrate how users can publish their data sets as interactive data visualizations with our new tools.

## Implementation

The exporter modules were developed for Cytoscape 3. The Cytoscape.js exporter is part of the Cytoscape 3 core distribution and is available as a standard feature. The D3.js exporter is an app, which means developers can write any type of additional JSON exporters as necessary. Exporter modules generate JavaScript Object Notation (JSON) files that are readable by Cytoscape.js and D3.js. To visualize these JSON files as interactive network diagrams, users have to write some JavaScript and HTML5 code to read, map, and render the network and table data in the files. The general structure of code for basic network visualization is common to most use cases. To simplify this visualization task, we provide template HTML5 projects to render the exported JSON files.

### Cytoscape.js exporter

Cytoscape.js is a JavaScript library for interactive network visualization developed by the Cytoscape Consortium. Although Cytoscape and Cytoscape.js share core concepts, they have completely independent code bases written in Java and JavaScript. This means there is no binary-level compatibility between these two software packages. The purpose of this exporter is to provide data-level compatibility between these software packages. There are two core functions in this exporter module, which are Cytoscape.js format JSON file generator from Cytoscape networks and tables, and Visual Style converter that creates Cytoscape.js compatible styles as JSON files.

### Network/Table to JSON converter

Conversion from Cytoscape networks and tables to Cytoscape.js JSON is done by a serialization module implemented with Jackson, a Java-based JSON parser library ( https://github.com/FasterXML/jackson). The converter takes a Cytoscape network object and associated node, edge, and network tables as inputs and converts them into a single JavaScript object represented as JSON. Most of the basic data types are converted into JSON except for nested networks, custom graphics, and node shapes and edge line types, which are only available in Cytoscape.

### Visual Style to CSS

**Figure 1.**
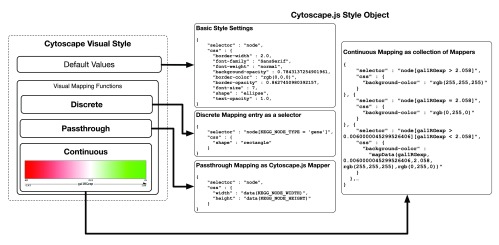
Cytoscape Visual Style to Cytoscape.js Style Object conversion.

In contrast, converting Cytoscape’s Visual Styles is a non-trivial process. A Visual Style in Cytoscape is a collection of visual mapping functions, which is a mapping from data to visual variables ^9^, and default values. Conversion from default visual property values to Cytoscape.js objects is a simple one-to-one mapping. However, some of the Cytoscape visual property values are not compatible with Cytoscape.js, and they will be filtered before the conversion. For example, in Cytoscape, there are three types of visual mapping functions. They are Passthrough, Continuous, and Discrete. Cytoscape.js has the concept of a visual mapping function in its design and it follows standards of CSS and selectors, which is significantly different from the design of Cytoscape visual mapping functions. The converter translates Cytoscape visual mapping functions into combinations of Cytoscape.js selectors and mappers ( Figure 1). This translation absorbs differences in design between the two applications and reproduces Cytoscape Visual Styles as JavaScript objects used for styling in Cytoscape.js.

In Cytoscape 3.1.1, some of the visual properties, such as node custom graphics and border line type, are not supported by this translator. Those properties will be supported in the future version of Cytoscape.

### D3.js exporter

In general, D3.js does not have any specific data format for visualization. Generic CSV/TSV tables can be used for all types of visualizations, and its core provides data loaders for those files. An exception is the graph data format for force-layout, which is the basic preset for visualizing graph data in D3.js ( https://github.com/mbostock/d3/wiki/Force-Layout). It uses an ordinal (i.e., zero-based) index of nodes as the unique identifier, and edges are represented as a pair of those indices. The D3.js exporter converts Cytoscape network topology into this force-layout format, and transforms all associated data tables into properties of nodes and edges in the JSON.

A tree data structure is a special kind of graph, and D3.js has various types of preset visualizations for it, such as radial layout, circle packing or Treemap. Cytoscape can visualize trees as node-link diagrams, and if we can export tree data models stored as Cytoscape graph objects into a D3.js compatible format, users can create multiple views for the same data sets using different D3.js visualization presets which could provide new insights for them. To utilize these presets, the exporter generates tree-style JSON for D3.js. The root node of the tree must be specified manually by the user, then the exporter automatically generates tree-style JSON with all associated tables.

Both of the export functions are available under Cytoscape’s *File* → *Export* → *Networks*... menu item.

### Template projects for visualizing JSON exports

To visualize the exported JSON files, actual web applications are needed to visualize the data. Both Cytoscape.js and D3.js are designed for developers, not for end-users, and developers are expected to write their own custom visualization code. Although they are optimized for custom web-based visualizations, basic components of visualization code, including data loading, mapping, and rendering, are common to most applications. To minimize duplicate efforts to visualize the results from JSON exporters, we developed template web application projects to visualize JSON files generated by Cytoscape. These templates can create basic visualizations of the JSON files out of the box. To develop these templates, we used standard tools for modern JavaScript development: Node.js ( http://nodejs.org/) as runtime for all development tools, Yeoman ( http://yeoman.io/) for code scaffolding, and Grunt ( http://gruntjs.com) as task runner.

## Results

**Figure 2.**
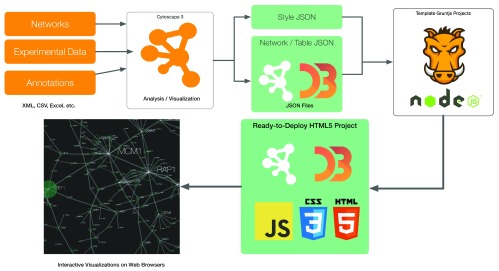
Basic workflow for publishing Cytoscape-generated networks as interactive web visualizations. The combination of Cytoscape 3 and the new JSON exporters can be used as a data integration tool for web-based visualizations. Our sample Grunt project generates a simple template code for visualizing D3.js and Cytoscape.js JSON files.

A typical data visualization workflow with our new tools consists of the following four steps. First, users load networks, annotations, and experimental data sets into Cytoscape. Second, utilizing core Cytoscape visualization features, users create custom Visual Styles and layouts. Third, users export all data sets as JSON files, and finally they can create custom web-based, interactive visualizations from the template projects ( Figure 2). The original network data visualized in Figure 5 was imported with a Cytoscape app called KEGGScape ( http://apps.cytoscape.org/apps/keggscape). The advantage of this workflow is that users can use the large collection of existing Cytoscape apps for data integration and analysis, and the result can be exported as interactive web-based visualizations with the new exporters. Figure 5 shows the TCA Cycle pathway which was generated from a KEGG XML (KGML) file, and its Visual Style was automatically generated from the graphics data in the file. Cytoscape.js exporter can generate web-compatible style and network files directly from the Cytoscape view. Our template code for Cytoscape.js is a simple viewer, and it can be used as a basis for complex data visualization application.

**Figure 3.**
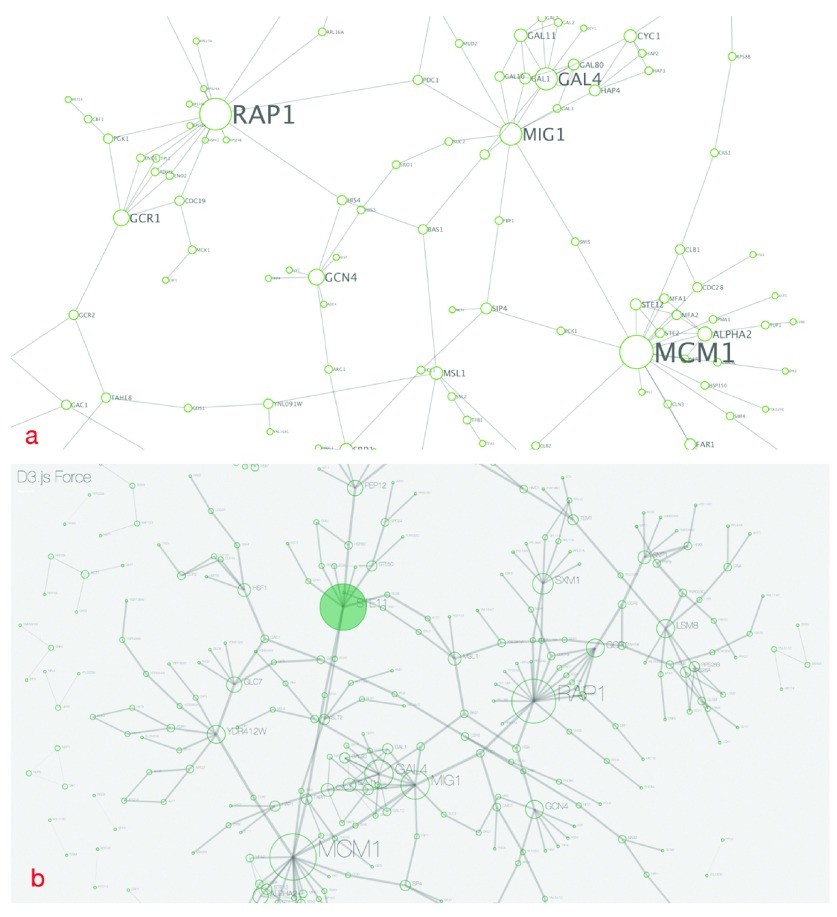
Visualization of a sample yeast protein-protein interaction network (galFiltered.sif from Ideker *et al.*, Science 292:929 2001. Available as a sample file in Cytoscape 3 distribution) by **a**) Cytoscape 3.1.1 and **b**) D3.js force-layout.

**Figure 4.**
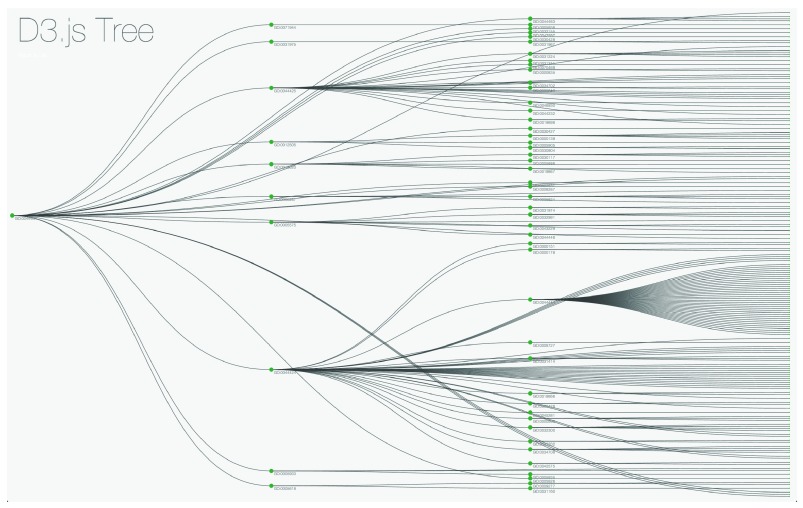
Tree version of Gene Ontology visualized by D3.js tree layout.

The exporters create JSON files with both network topology and data tables, and users can create complex data visualizations which cannot be achieved with Cytoscape alone. Figure 3 and Figure 4 shows simple network visualizations created with the D3.js force-directed layout ( Figure 3) and tree layout ( Figure 4). These figures are created with a minimal set of features available in D3.js and they can be used as a “boilerplate” code for custom visualizations. The desktop version of Cytoscape is optimized for rendering node-link, or ball-stick network diagrams, which is only one way to visualize graph data while Cytoscape 3 supports multiple-rendering engines and if developers can implement new rendering engines for new visualizations, such as Treemaps or Chord Diagrams, they can add new visualizations on Cytoscape. This is not a trivial task and as an alternative, our D3.js templates can be used to make prototypes for new visualizations.

**Figure 5.**
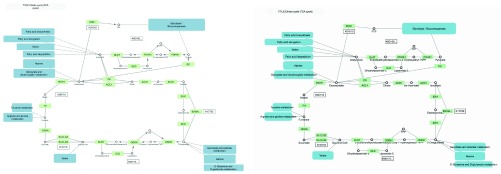
Original network view in Cytoscape 3.1 (left) and same data exported as JSON file and visualized by the template code using Cytoscape.js (right). Original network data was imported to Cytoscape 3 using the KEGGScape app. Cytoscape.js version reproduces almost all details from the original Cytoscape network view. Step-by-step instruction to create web visualization is available at https://github.com/idekerlab/cyjs-exportparent/wiki.

## Conclusions

In this paper, we presented a new workflow to visualize biological data sets using Cytoscape and modern web-based data visualization libraries. The example visualizations show how users can leverage easy-to-use Cytoscape core features as a part of web-based interactive data visualization publishing workflow.

### Future plan

At this point, new features discussed in this paper are designed for developers who can write JavaScript and HTML5 code. End users are also a part of our target audience, and so we will implement the “Export as HTML5 Session” feature as a core Cytoscape feature, which creates a compressed archive file that includes all of the networks, tables, and Visual Styles as JSON along with all JavaScript files to visualize the data as a single-page application.

### Software availability

**Software available from:**

http://apps.cytoscape.org/apps/d3jsexporter

**Latest source code:** https://github.com/keiono/cytoscape-d3

**Source code as at the time of publication:**

https://github.com/F1000Research/cytoscape-d3/releases/tag/V2.0

**Archived source code as at the time of publication:**

http://dx.doi.org/10.5281/zenodo.12224 ^10^

**License:** MIT License

**Template web application code for Cytoscape.js:**

https://github.com/idekerlab/cyjs-export-parent

**Template web application code for D3.js Exporter:**

https://github.com/keiono/d3-exporter-sample

High-resolution images and interactive examples are available at our web sites above.
