# Supplementary material for: Cytoscape tools for the web age: D3.js and Cytoscape.js exporters
Source: F1000Res. 2014 Oct 28;3:143. Originally published 2014 Jul 1. [Version 2] doi: 10.12688/f1000research.4510.2 (PMC4264639; doi:10.12688/f1000research.4510.2)
Supplement: Peer review history

# Peer review: Referee response for version 2

**DOI:** 10.5256/f1000research.5911.r6525

**Published:** 2014-12-01

**Authors:** Alexander Lex

**Affiliations:** 1 Visual Computing Group, Harvard University, Cambridge, MA, USA

Dear authors,

Thank you for your revision. My comments on the initial version are completely addressed and I do approve of the current version. 

Here are some minor suggestions for improvements of the text: 

- To visualize the exported JSON files, actual web applications are needed to visualize the data.  -> To visualize the exported JSON files, actual web applications are needed.
- This sentence should probably be split into two:
  The desktop version of Cytoscape is optimized for rendering node-link, or ball-stick network diagrams, which is only one way to visualize graph data while Cytoscape 3 supports multiple-rendering engines and if developers can implement new rendering engines for new visualizations, such as Treemaps or Chord Diagrams, they can add new visualizations on Cytoscape.
- Step-by-step instruction to create web visualization is available at ->  Step-by-step instructions to create the web visualization are available at

I have read this submission. I believe that I have an appropriate level of expertise to confirm that it is of an acceptable scientific standard.

# Peer review: Referee response for version 1

**DOI:** 10.5256/f1000research.4824.r6061

**Published:** 2014-09-11

**Authors:** Jose Villaveces

**Affiliations:** 1 Research Group Computational Biology, Max Planck Institute of Biochemistry, Munich, Germany

The article describes Cytoscape.js and D3.js exporters and a code template generator. Additionally, it provides all the essential information about Cytoscape.js exporter and D3.js exporters and points interested users to the source code for its implementation.

Even though there are a few points that require clarification, the tools are a nice addition to Cytoscape and can be seen as a first step in uniting present day web technologies and the more conventional desktop based visualisation provided by Cytoscape.

**Improvements**​

1. There is no documentation for the template generator, a README.md file with a few examples will be nice.
2. The D3 example listed in the d3jsexporter page is very small (300 x 150) and the 'View Source' button is not working.

I have read this submission. I believe that I have an appropriate level of expertise to confirm that it is of an acceptable scientific standard.

# Peer review: Referee response for version 1

**DOI:** 10.5256/f1000research.4824.r5313

**Published:** 2014-08-01

**Authors:** Alexander Lex

**Affiliations:** 1 Visual Computing Group, Harvard University, Cambridge, MA, USA

The article describes an important new development for the Cytoscape project: bridging the gap between complex yet powerful desktop applications to easy-to-use, installation free web application focused on the analysis and presentation. I consider this workflow as an important bridge between the two worlds. The article is technically sound and mostly well written. Some structural comments and concrete suggestions for improving the manuscript are listed below. 

What I am currently missing in the manuscript is a description of a workflow for cleaning up the exported visualizations. Especially in the KEGG example, the result is rather messy and clearly requires some additional authoring. How would one go about this process? How long would it take? I would suggest adding another figure that shows a post-cleanup version and a paragraph commenting on the process. 

Also, it would be great to see a comparison of the original layout in Cytoscape in addition to the web-based layouts, ideally for all the results figures, so that readers can clearly judge the quality of the approach. This could also put my previous comment into context, as maybe the KEGG layout isn't much better in Cytoscape standalone, since the KEGG annotations typically don't contain sufficient information about the layout.

Structural Suggestions:

- For this argument: 
*"However, since they were designed before the re-discovery of Ajax, developers could not predict the success of JavaScript and related web technologies today."*
  I would suggest to add that it was simply not feasible at the time since the technology and bandwidth was not available.
- Regarding this sentence:
*"It is hard to perform exploratory data analysis only with static images, especially when visualizing large data sets, because some of the details are lost due to the limited size of printed papers or computer screens."*
  I would suggest to also argue that this static approach is especially problematic for networks, as the size of all but trivial networks requires dynamic features to be useful. 
- Here: 
*"If biologists can use the existing powerful desktop application and these emerging web technologies for data visualizations and sharing, it opens up a new way to understand large and complex biological data sets."*
  I think the main point is that it has the potential to combine the best of two worlds: powerful authoring tools and easily shareable interactive results, but I'm not sure whether this is "a new way to understand[ing]". Rather it is a new way of engaging users, through a much simplified process.
- *"via a web platform (e.g., browser)"* -> what would be other web platforms? Or do you mean *"i.e., in the browser"*
- The hierarchy in the Cytoscape.js exporter section isn't clear. The following two captions seem to be of the same level as the root section. I suggest removing the captions completely and simply make the converters bold in the text. Also, these sections sometimes lack clarity. For example, it is not clear what a Cytoscape Table and Network is. I assume that is clear when one knows the software well, but it would be good to clarify this here for readers who are not that familiar with Cytoscape.
- What are *"non-compatible value filterings"*?
- *"The root node of the tree should be specified manually by the user"* -> should or must?
- Caption Figure 4 *: "Visualization of a sample network (galFiltered.sif) by D3.js force-layout."* -> What is the data shown here? Should a reader know that from the file name? The file name seems irrelevant here. I would rather add a brief description of the data. 

Some grammar and style suggestions:

- *"to popular JavaScript library readable formats"* -> to *"formats readable by popular JavaScript libraries.".*
- *"and Java-based feature-rich toolkits were developed"* -> " *and many Java-based feature-rich toolkits were developed" .*
- *"technologies, Cytoscape Consortium"* -> " *technologies, the Cytoscape Consortium".*
- *"libraries (matplotlib, ggplot2)"* -> " *libraries (e.g., matplotlib, ggplot2)".*
- *"The goal is to enable shared visualization of Cytoscape networks via a web platform (e.g., browser), and our strategy is to enable conversion from Cytoscape data objects to a format friendly to web apps, and to provide a template code for creating an interactive web application." *-> split into multiple sentences.
- *"is part of Cytoscape 3 core distribution"* -> *"is part of the Cytoscape 3 core distribution".*
- *"The D3.js exporter is an app, and this means"* -> " *The D3.js exporter is an app which means"*
- *"The D3.js exporter is an app, and this means"* -> I suggest clarifying " *app"*. Is this a cytoscape plugin?
- *"we implemented template HTML5 projects to render the exported JSON files."* -> *"we provide template HTML5 projects to render the exported JSON files.".*
- *"developed by Cytoscape Consortium"* -> *"developed by the Cytoscape Consortium".*
- *"There are two core functions in this exporter module: network/table converter and Visual Style to CSS converter."* -> I would suggest rephrasing this to explain on a high level what these modules do.
- *"except the following: nested networks,"*  -> " *except for nested networks,".*
- "c *onverting Cytoscape Visual Style"* -> is this a noun? or do you mean *"Cytoscape's visual styles". *
- In Figure 1, the code in the *"Continuous Mapping as collection of Mappers"* is unreadable as it is too small.
- *"Once exporters generate JSON files, users need actual web applications to visualize the data."* -> *"To visualize the exported JSON files, actual web applications are needed to visualize the data."*.
- *"visualization code to see the data."* -> *"visualization code."*
- *"Third, all data sets are exported as JSON files, and finally they can create custom web-based, interactive visualizations from the template projects (Figure 2)."* -> This sentence mixes active and passive. I suggest re-writing it in the active voice since the previous sentence is active as well.
- *"The combination of Cytoscape 3 and new JSON exporters can be used as data integration tool for web-based visualizations"* -> *"The combination of Cytoscape 3 and the new JSON exporters can be used as a data integration tool for web-based visualizations.".*
- *"created with D3.js force-directed layout"*  -> *"created with the D3.js force-directed layout". *
- *"such as Treemap or Chord Diagram"* -> " *such as Treemaps or Chord Diagrams".*
- *"License MIT License"* -> " *License: MIT License".*
- *"Template code generator website: https://github.com/cytoscape/cyjs-sample"* -> *"Template code generator website https://github.com/cytoscape/cyjs-sample".*

I have read this submission. I believe that I have an appropriate level of expertise to confirm that it is of an acceptable scientific standard, however I have significant reservations, as outlined above.

# Response

**Published:** 2014-08-06

**Authors:** Keiichiro Ono

**Affiliations:** University of California, San Diego, USA

I completely agree with your point about the missing details for web based KEGG visualization part.  I will add more details how to reproduce the visualization with this tool.  It will be available as a GitHub Wiki page.  The following details will be added to the new version:

- Side-by-side comparison of original Cytoscape visualization and exported version
- Original Cytoscape Session file and exported JSON files as supplemental data
- Link to the instruction wiki page how to reproduce the KEGG visualization

In addition to these changes, I will fix the structural/grammatical issues in the revised version.

Thank you for your helpful suggestions.

# Response

**Published:** 2014-11-25

**Authors:** Keiichiro Ono

**Affiliations:** University of California, San Diego, USA

Dear Dr. Lex,

This version 2 includes all updates for your comments for the original version.  Also, recently we released new version of Cytoscape (3.2.0), and it should be compatible with this protocol.  If you have problems to test this workflow in the latest environment, please let me know.

Best,

Keiichiro Ono
